# Aneurism after Venesection

**Published:** 1830-04-01

**Authors:** 


### HOPITAL DE LA CHARITE.

### Circumscribed Aneurism after Venesection—Cure from the Hunterian Operation.

It is strange to see what a superstitious kind of horror is attached by many medical men to the puncture of the brachial artery in the operation of venesection. When the accident has taken place the parties appear generally to lose their wits, and fly to all kinds of injudicious measures. We remember witnessing a case of this kind, in which a tourniquet had been thrust upon the limb and allowed to remain for one or two days, with the effect of producing the most excessive and injurious tumefaction. There are instances enough upon record to prove that moderate and *properly applied* pressure will frequently effect a cure of the puncture, and even if it fails it may perhaps reduce the case to one of false aneurism, to be treated like false aneurism elsewere by the Hunterian operation.

*Case.**—Virginia Dooux, twenty-three years of age, was bled in August 1829, for some nervous symptoms under which she laboured. At the instant of the operation she felt a kind of tremor running to her fingers and the nature of the jet discovered that the artery was wounded. The practitioner allowed a certain quantity of blood to escape, and bandaged the limb very firmly, but without entirely arresting the hæmorrhage. The limb swelled greatly and the bandage was removed next morning, when although the puncture was not closed, no bleeding took place. The swelling of the arm remained for five days with pain and inflammation round the wound, a sensation of a constant beating was experienced at the bend of the elbow, but in the course of a week the limb admitted of extension. In the course of another week a small pulsating tumour, in size resembling a filbert, made its appearance, increased with rapidity, was treated unsuccessfully by the application of ice, and was so far advanced at the end of five weeks that the patient entered the hospital of La Charité.

At this time, Sept. 7, the tumour was rounded, about an inch in length, situated immediately above the fold of the arm on the inside of the tendon of the biceps. The skin covering it was not changed in colour, it pulsated visibly in synchrony with the pulse at the wrist, and not only in the tumour but also throughout the artery a peculiar whizzing sensation was communicated to the finger. On applying the stethoscope in the same situations, a distinct *bruissement* was perceptible.

---

* Journ. Hebdomad. No. 59.

On compressing the brachial artery about the middle of the arm the size of the tumour was greatly diminished; no varicose vein existed at the bend of the arm, but several large veins ramified beneath the skin on the outside of the tumour; the hand and fore-arm were free from swelling, the motions not materially impaired. The patient had been married about a year, was troubled with mænnorrhagia, and suffered from those various nervous pains so frequently attendant on disordered menstruation.

At first it was suspected that the case might be one of varicose aneurism, but the examination and the facts above elicited proved, beyond a doubt, that it was one of consecutive false aneurism.* On the 12th of Sept. M. Roux performed the operation of tying the artery above the tumour, in accordance with the principle adopted in the treatment of false aneurism elsewhere. The patient being laid upon a bed with the right arm conveniently placed, the course of the brachial artery was ascertained by the finger, and an incision fifteen lines in length was made towards the middle of the arm, and on the inner side of the biceps muscle. A vein which was divided in this incision, bled smartly. The fascia and cellular sheath of the brachial artery and median nerve having been divided, a grooved director, slightly curved at its termination, was introduced from within outwards beneath what appeared to be the artery, and a probe armed with a double thread was passed along the groove. The probe was withdrawn and the ends of the threads raised, but without the effect of arresting the pulsations of the tumour. On the contrary the patient experienced only intense suffering, and it was evident enough that the nerve and not the artery was engaged. The latter was discovered deeper, though raised by the director; the threads were withdrawn from the nerve and passed by means of the probe around the artery; their ends were then raised with the effect of commanding the circulation in the tumour; all was now right, and accordingly the ligatures were tied according to Scarpa's method, on a cylinder of *sparadrap* applied upon the vessel. The tumour immediately diminished in size, the pulse ceased in the radial artery, and the wound was dressed to the bottom with lint and covered with compresses and roller. The limb was enveloped in warm rags, and placed on a cushion in the semi-flexed position.

A little heat of skin and numbness of the fingers took place during the day, and a bad night with fever ensued. The hand was warm and retained so by means of heated cloths, in addition to which an anodyne draught and a lavement were prescribed. Next day the patient was much better, but there was still some numbness in the fingers and particularly in the thumb, and at the patient's request a smaller bandage and compress were applied. The tongue was loaded, the belly slightly tender on pressure, and the bowels not opened, but otherwise the general health was good. A lavement and anodyne were again exhibited, but without effect *quoad* the bowels, in consequence of which calomel was exhibited on the 15th and castor oil in lavement on the 16th, when copious evacuations and complete relief were the consequence. Suppuration of the wound took place, pulsation began to return in the radial artery on the 17th, the ligatures came away on the 26th, and the patient left the hospital cured in the early part of October. The tumour was at this time quite solid, and reduced to the size of a very small pea; the motions of the fore-arm on the arm were still difficult, especially those of extension; and a slight degree of numbness was occasi-

* By consecutive false aneurism is meant an aneurismal sac formed from the cellular membrane, and communicating with the interior of the artery by means of the wound in the latter. We recommend such of our readers as are not perfectly acquainted with the modern nomenclature of aneurismal affections, to study the admirable work of Mr. Hodgson on the Diseases of the Arteries. It is a work which ought to be in the hands of every one.—Ed.

onally felt in the fingers. She was recommended on her departure to use local baths of gelatine, and *douches* directed on the arm.

This is a good specimen of the success that occasionally attends the operation of tying the artery above the tumour in the circumscribed aneurism after wound of the vessel in bleeding. In the diffused aneurism, that in which the blood is extensively effused into the cellular membrane, and an operation is not to be recommended, but the surgeon must cut down and tie the vessel above and below the puncture, as in any other case of wounded artery. Even in circumscribed aneurism like the present, when a sac is formed from the cellular membrane, the Hunterian operation will occasionally fail in consequence of the free ingress of blood from below, and it then becomes necessary to tie *all* the branches entering the sac, in fact to have recourse to the old operation for aneurism. A very interesting case of this kind was published by Mr. Brodie a few years ago, and noticed in this Journal. If before proceeding to the operation it is found that pressure on the brachial artery above the tumour exercises a decided and uncontrolled influence on its pulsation and size, the probability is that one ligature in that situation will answer. If it does not arrest the pulsation, or doing that has no effect on the bulk of the tumour, the probability is then just the other way, and the surgeon must lay his account to the Hunterian operation failing of success. The subject is one of such extreme interest that we feel great reluctance in waiving the consideration of many other questions connected with it.

